# Overexpression of Arabidopsis *OPR3* in Hexaploid Wheat (*Triticum aestivum* L.) Alters Plant Development and Freezing Tolerance

**DOI:** 10.3390/ijms19123989

**Published:** 2018-12-11

**Authors:** Alexey V. Pigolev, Dmitry N. Miroshnichenko, Alexander S. Pushin, Vasily V. Terentyev, Alexander M. Boutanayev, Sergey V. Dolgov, Tatyana V. Savchenko

**Affiliations:** 1 Institute of Basic Biological Problems RAS, Pushchino 142290, Russia; alexey-pigolev@rambler.ru (A.V.P.); miroshnichenko@bibch.ru (D.N.M.); aspushin@gmail.com (A.S.P.); v.v.terentyev@gmail.com (V.V.T.); boutanaev@mail.ru (A.M.B.); 2 Branch of Shemyakin and Ovchinnikov Institute of Bioorganic Chemistry RAS, Pushchino 142290, Russia; dolgov@bibch.ru

**Keywords:** transgenic wheat, 12-oxophytodienoate reductase, jasmonates, freezing tolerance

## Abstract

Jasmonates are plant hormones that are involved in the regulation of different aspects of plant life, wherein their functions and molecular mechanisms of action in wheat are still poorly studied. With the aim of gaining more insights into the role of jasmonic acid (JA) in wheat growth, development, and responses to environmental stresses, we have generated transgenic bread wheat plants overexpressing *Arabidopsis 12-OXOPHYTODIENOATE REDUCTASE 3* (*AtOPR3*), one of the key genes of the JA biosynthesis pathway. Analysis of transgenic plants showed that *AtOPR3* overexpression affects wheat development, including germination, growth, flowering time, senescence, and alters tolerance to environmental stresses. Transgenic wheat plants with high *AtOPR3* expression levels have increased basal levels of JA, and up-regulated expression of *ALLENE OXIDE SYNTHASE*, a jasmonate biosynthesis pathway gene that is known to be regulated by a positive feedback loop that maintains and boosts JA levels. Transgenic wheat plants with high *AtOPR3* expression levels are characterized by delayed germination, slower growth, late flowering and senescence, and improved tolerance to short-term freezing. The work demonstrates that genetic modification of the jasmonate pathway is a suitable tool for the modulation of developmental traits and stress responses in wheat.

## 1. Introduction

Plant growth, development and responses to environmental signals are coordinated by complex multicomponent signaling networks. Jasmonates, derived from fatty acids, are important components of this regulatory system. Jasmonates biosynthesis and signal transduction pathways have been thoroughly studied in model plant Arabidopsis [1,2,3,4]. The biosynthesis is initiated in chloroplasts, where lipoxygenases oxidize the 13th carbon atom of the α-linolenic acid, forming 13(S)-hydroperoxy-9,11,15-octadecatrienoic acid (13-HPOT). The 13-HPOT is further converted into (9S,13S)-12-oxo-phytodienoic acid (12-OPDA) by the consequent action of allene oxide synthase and allene oxide cyclase, and then 12-OPDA is transported to peroxisomes. Inside the peroxisomes, the double bond of the cyclopentenone ring in 12-OPDA is reduced by 12-OPDA reductase [5], and three cycles of β-oxidation of the carboxylic acid side chain occur, resulting in the formation of JA. JA itself can be further modified in the cytoplasm through the formation of a conjugate with the amino acid isoleucine, the signaling ligand, which is responsible for the regulation of majority of JA-dependent processes. Several metabolites of the pathway, 12-OPDA, JA, jasmonate–isoleucine conjugate, and methyl jasmonate display biological activity with only a partial overlap in functions. To date, most of the information on jasmonates biosynthesis and signaling comes from studies of dicotyledonous species, mainly Arabidopsis [1,2,3,4,5]. In monocots, this pathway is less well studied, and the information is mainly limited to maize and rice [6]. Biosynthesis, functions, and molecular mechanisms of action of jasmonates in wheat, one of the dominant cereal crops worldwide, remain mostly uncharacterized.

Unfavorable environmental conditions affect wheat crop yield globally [7]; therefore, the improvement of wheat tolerance to environmental stresses is of particular importance. The modification of individual steps of oxylipin biosynthesis pathway results in the alteration of plant tolerance to environmental challenges, as shown for Arabidopsis [8,9,10,11,12] and other species, including a few monocots [6,13,14]. For wheat, a protective effect of exogenously applied jasmonates under conditions of biotic and abiotic stresses was demonstrated in numerous studies [15,16,17], suggesting that the modulation of the activity of jasmonate-based signaling systems is a promising approach for the improvement of wheat tolerance to environmental stresses. The effect of the genetic modification of the jasmonate pathway on wheat development and stress response has not been sufficiently investigated, probably due to the fact that hexaploid wheat remains recalcitrant in tissue culture [18]. With the purpose of altering the level of jasmonic acid and studying the effect of such alterations on plant growth, development, and stress responses, we have generated transgenic hexaploid (*Triticum aestivum* L.) wheat plants constitutively overexpressing the *12-OXOPHYTODIENOATE REDUCTASE 3* gene from *Arabidopsis thaliana* (*AtOPR3*). 12-Oxophytodienoate reductases are classified into OPRI and OPRII subgroups, and only OPRII subgroup genes take part in the biosynthesis of jasmonic acid. Out of three oxophytodienoate reductases in Arabidopsis, only *OPR3* belongs to the OPRII subgroup, and it is involved in the jasmonate biosynthesis pathway [19]. *AtOPR3* is a well-characterized gene, coding for the enzyme controlling the levels of major metabolites of the jasmonate pathway, the upstream substrate 12-OPDA, and downstream products of the biosynthetic pathway, JA, and jasmonic acid derivatives [5,12,20,21]. The role of *AtOPR3* in plant growth and stress responses has been demonstrated [5,22,23], and the crystal structure of the OPR3 protein has been determined [24]. 

## 2. Results

### 2.1. Generation of Wheat Plants Overexpressing *AtOPR3*

**Figure 1:**
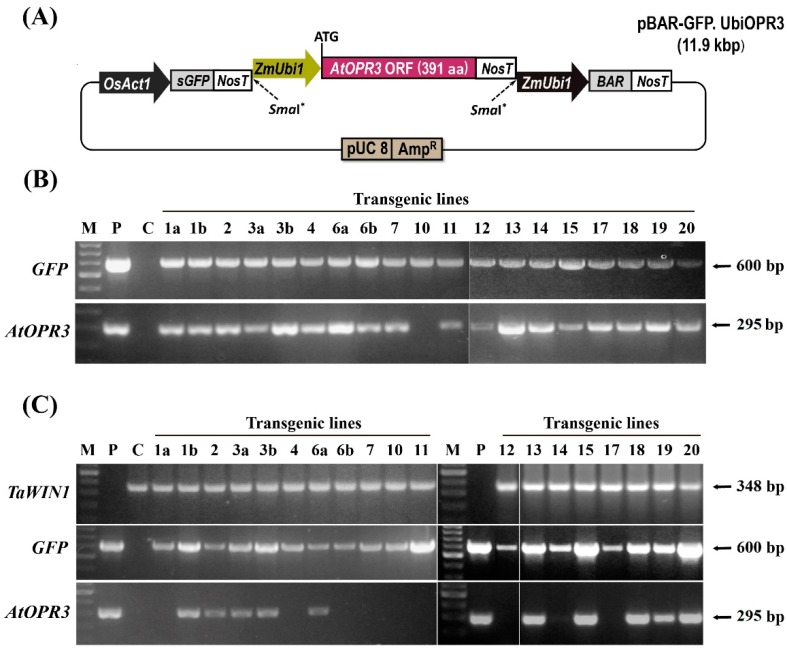
Generation and molecular analysis of transgenic plants. (**A**) Schematic representation of the pBAR-GFP.UbiOPR3 expression cassette used for Sar-60 transformation. *OsAct1*, rice Actin 1 promoter; *ZmUbi1*, maize ubiquitin 1 promoter; *NosT*, nopaline synthase terminator; *GFP*, Green Fluorescent Protein gene; *BAR*, BASTA resistance gene (phosphinothricin acetyl transferase); *AmpR*, ampicillin resistance gene. (**B**) PCR analysis performed on the genomic DNA of T0 plants for the insertion of *GFP* gene (upper panel) and *AtOPR3* (bottom panel). (**C**) End-point RT-PCR analysis on the total RNA of T0 plants (at heading stage) for the expression of the reference gene *TaWIN1* (top panel), *GFP* gene (middle panel) and *AtOPR3* (bottom panel). Lane M, DNA ladder as a molecular weight marker; Lane P, plasmid DNA pBAR-GFP.UbiOPR3; Lane C, non-transgenic wheat plant Sar-60; Lanes Tr-1–Tr-20 represent putative transgenic wheat plants.

The hexaploid spring bread wheat variety ‘Saratovskaya-60’ (Sar-60) was previously thoroughly characterized and used as a parent line for the generation of a number of commercial wheat varieties grown in Russia [25]. In the present study, Sar-60 was genetically transformed with the pBAR-GFP.UbiOPR3 vector, enabling the expression of the *Arabidopsis thaliana OPR3* gene under the control of a strong constitutive promoter (Figure 1A). A number of transgenic plants of various generations (T0–T3) were produced to study the effect of *AtOPR3* overexpression in wheat. A total of 23 putative transgenic plantlets produced from 20 independent explants, designated as Tr-1 to Tr-20, were established in the greenhouse. In a few cases, two plants were regenerated from one explant (1a and 1b, 3a and 3b, 6a and 6b). Sixteen primary independent lines were identified as being transgenic by PCR, with a pair of primers targeting a 600 bp fragment of *GFP* (Figure 1B, upper panel), and a 295 bp fragment of *AtOPR3* (Figure 1B, lower panel). *AtOPR3* was detected in all transgenic lines, except line 10. End-point RT-PCR was performed on leaf samples collected from plants at the heading stage, to confirm the expression of the introduced genes in *GFP*-positive primary transgenic lines. The *TaWIN1* gene [26] was used as a control for the expression analysis (Figure 1C, upper panel). The *GFP* transcript was detected in all studied plants except for non-transformed Sar-60 control (Figure 1C, middle panel). The amplification bands corresponding to *AtOPR3* were specifically detected in the nine primary transgenic lines, but not in non-transformed Sar-60 plants and several green fluorescent protein (GFP)-positive transgenic lines (Figure 1C, lower panel). 

T0 plants from eight *AtOPR3*-positive events produced T1 seeds, and segregation analysis was conducted on their progeny via GFP fluorescence, as described in [27]. The embryos produced by self-pollination of individual T1 plants were used for the detection of homozygous populations. At first, we analyzed the pollen produced by T1 plants, collected immediately after the anthers appeared (Figure S1A). If the pollen displayed a homozygous pattern, T2 zygotic embryos were additionally analyzed for GFP fluorescence (Figure S1B). Using this method, we were able to identify homozygous offspring, derived from six self-pollinated independent primary (T0) lines. The successful expression of transgenes in homozygous plants of the selected T2 population was confirmed by end-point RT-PCR (Figure S1C). Homozygous lines with confirmed expression of *AtOPR3* in the T2 generation, which produced sufficient amount of seeds, including Tr-3 (the progeny from 3b line), Tr-15, Tr-18, Tr-19, and Tr-20, were used for further analysis.

### 2.2. Transgenic Wheat Plants Overexpressing the Arabidopsis *OPR3* Gene Display an Altered Growth and Development Phenotype

**Figure 2:**
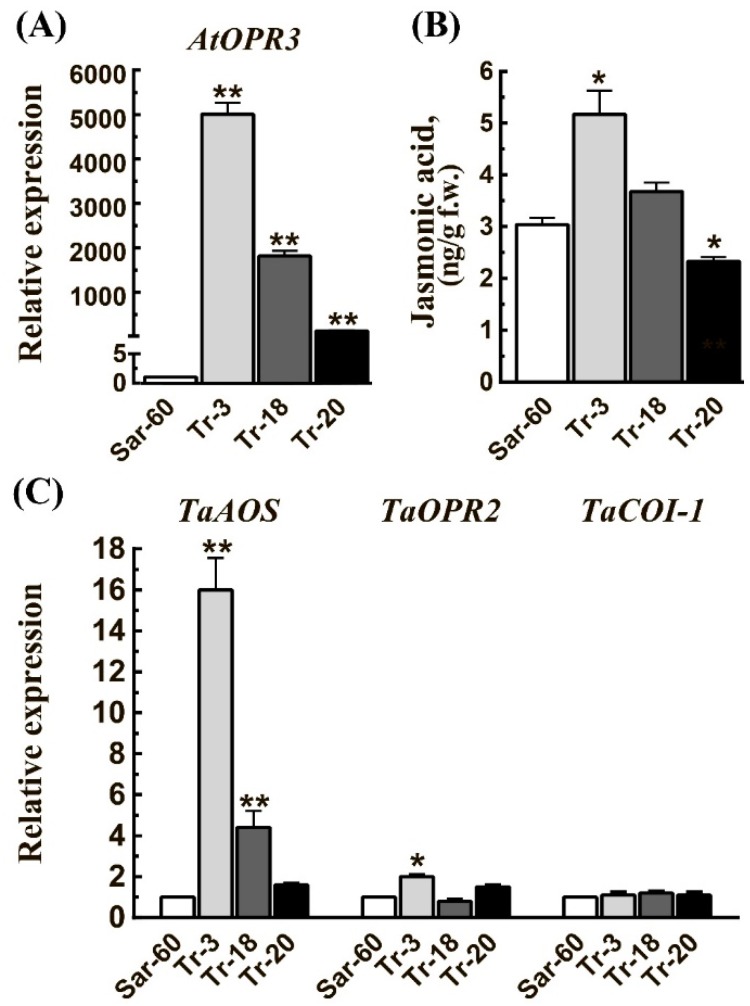
Gene expression analysis and JA levels in transgenic plants. The relative expression of *AtOPR3* (**A**), and endogenous wheat jasmonate biosynthesis and signaling pathways genes *TaAOS*, *TaOPR2*, and *TaCOI-1* (**C**). Total RNA was extracted from leaves (the second leaf of T3 plants at four leaves stage) and subjected to real-time quantitative RT-PCR analysis. The transcript levels of each gene were normalized to *TaWIN1* and *TaUbi*, as measured in the same samples. Data are means of four biological replicates represented by 6–8 leaves ± SE. (**B**) JA levels in second leaves of T3 plants collected at the four-leaf stage. Stars above the graphs indicate statistically significant differences (* *p* ≤ 0.05, ** *p* ≤ 0.001).

**Figure 3:**
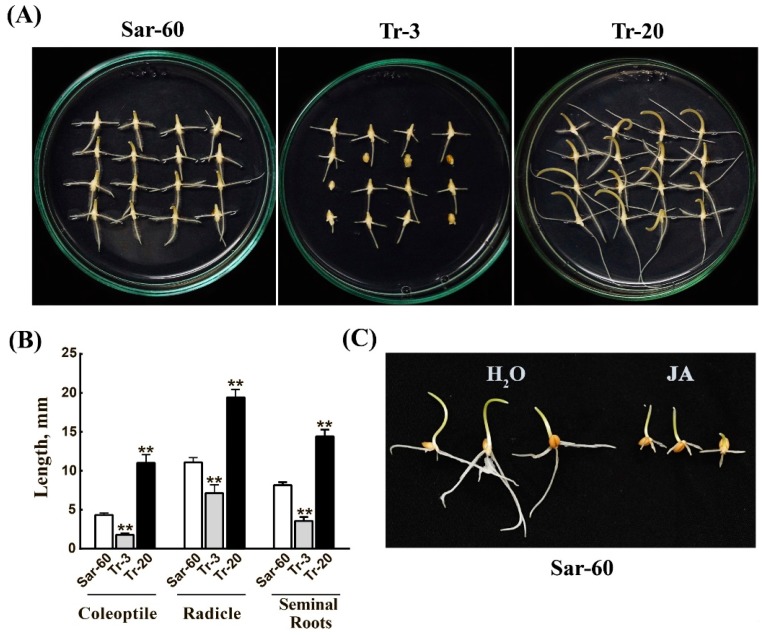
Jasmonate suppresses germination and early growth of wheat. (**A**) The representative Petri plates with seedlings grown from immature embryos of Sar-60, Tr-3, and Tr-20 T3 seeds, four days after culture initiation. (**B**) Length of coleoptiles, radicle and lateral seminal roots of four-day-old seedlings of Sar-60 (white bars), Tr-3 (grey bars), and Tr-20 (black bars). The means of 16 measurements ±SE are presented; stars above the graphs indicate statistically significant difference between genotypes (*p* ≤ 0.001). (**C**) Effects of exogenously applied MeJA on the germination and growth of Sar-60 seeds. Seeds were placed on filter paper soaked in distilled water (left side) or a 0.1 mM solution of MeJA (right side); the picture was taken after four days of seed imbibition.

**Figure 4:**
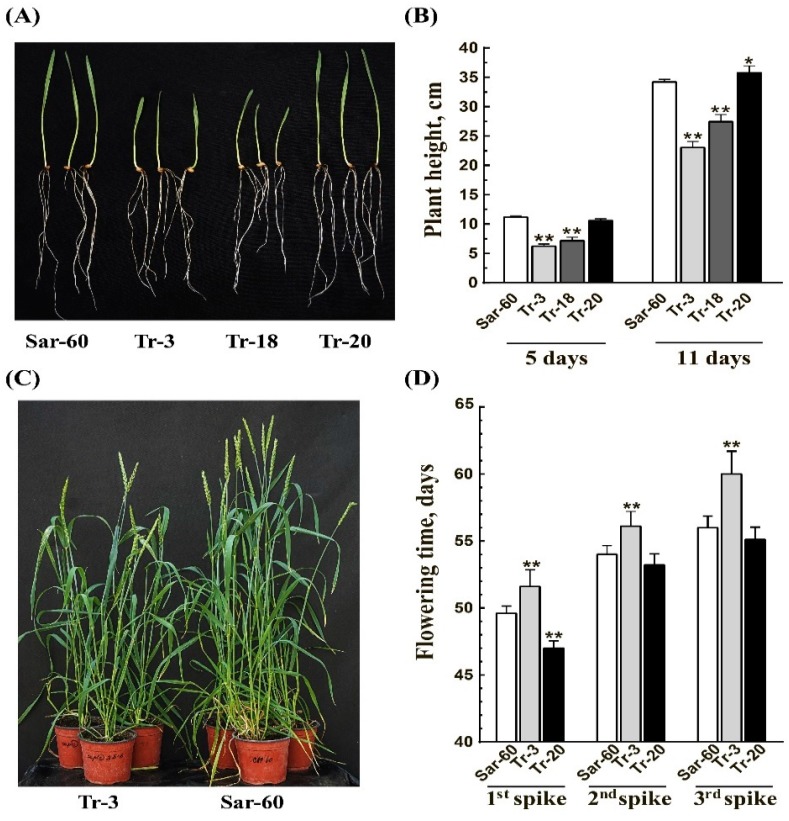
Transgenic wheat plants overexpressing *AtOPR3* gene have altered growth and flowering time phenotype. (**A**) The picture of soil-grown five-day-old homozygous T2 plants, Sar-60, Tr-3, Tr-18, and Tr-20. (**B**) The height of plants in 5 and 11 days after sowing. Means of 25 measurements ± SE are presented; stars above graphs indicate statistically significant differences (* *p* ≤ 0.05, ** *p* ≤ 0.001). (**C**) 55-day-old plants, Tr-3 and Sar-60. (**D**) Time (in days) of appearance of first, second, and third spikes on Sar-60 (white bars), Tr-3 (grey bars), and Tr-20 (black bars) plants. Means of 45 plants analyzed in three independent experiments ±SE are presented. Stars above the graphs indicate statistically significant differences (*p* ≤ 0.001).

**Figure 5:**
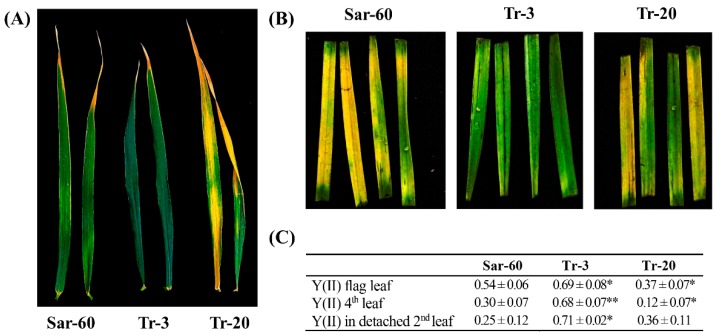
The timing of senescence is altered in wheat plants overexpressing *AtOPR3.* (**A**) Representative flag leaves of two-month-old homozygous T2 plants, Sar-60, Tr-3, and Tr-20. (**B**) The senescence of detached leaves. Same-aged detached leaves of Sar-60, Tr-3, and Tr-20 were kept floating on distilled water in Petri dishes under photoperiodic illumination 16/8 h (light/dark). The picture was taken on the fifth day of the experiment. (**C**) Analysis of PS II operating efficiency in flag (upper line) and fourth (middle line) leaves of two-month-old Sar-60, Tr-3, and Tr-20 plants growing in the soil, and detached leaves kept for five days under photoperiodic illumination (bottom line). Means of 13–16 measurements ± SE are presented. Stars indicate statistically significant differences from Sar-60 (* *p* ≤ 0.05, ** *p* ≤ 0.001).

To find out if there is an effect of *AtOPR3* gene overexpression on wheat plants growth and development, we carefully examined the germination, growth, flowering time, and senescence of generated transgenic lines. The observation allowed us to identify two distinct phenotypes among stable transgenic lines. The lines Tr-3 and Tr-18 (and to a lesser extent Tr-19) were characterized by delayed seed germination, slower growth, delayed flowering, and senescence, while the plants of Tr-15 and Tr-20, on the contrary, are characterized by earlier germination, faster growth, earlier flowering, and senescence (Figure 3, Figure 4 and Figure 5, and Figures S2–S4). Quantitative analysis of *AtOPR3* gene expression in T3 homozygous plants revealed a significant difference in the expression levels between transgenic lines (Figure 2A). *AtOPR3* expression level was highest in the Tr-3 line; in Tr-18, this level was lower by almost three times in comparison to Tr-3, and in Tr-20, it was lower by 40 times. The JA level was also highest in Tr-3 and lowest in Tr-20 and Tr-15 (Figure 2B). Interestingly, JA levels in Tr-20 and Tr-15 were decreased, even in comparison to Sar-60. The JA level in Sar-60 leaves was about 3 ng/g fresh weight; in Tr-3, this value was increased by 70%, in line Tr-18 the increase was about 21%, in lines Tr-20 and Tr-15 JA level was decreased by 23% (Table S1). We also analyzed the expression levels of three genes involved in jasmonates biosynthesis and signaling, *ALLENE OXIDE SYNTHASE* (*TaAOS*) (GenBank: KJ001800.1), *12-OPDA REDUCTASE 2* (*TaOPR2*) (TraesCS7B02G311600), and *CORONATINE INSENSITIVE 1-LIKE* (*TaCOI*-1) (TraesCS1A02G279100) (Figure 2C). The analysis revealed a significant increase in the level of *TaAOS* gene expression in Tr-3 (16-fold increase) and Tr-18 (4-fold increase) in comparison to Sar-60. Expression level of *TaOPR2* was slightly increased in Tr-3, while *TaCOI-1* levels remained unchanged in all analyzed lines.

We examined germination and early growth of immature embryos of Tr-3 and Tr-20, the lines with pronounced phenotype on the synthetic medium. The analysis showed that Tr-3 embryos germinated one day later than non-transformed Sar-60, while Tr-20 germinated one day earlier. The representative Petri plates with seedlings (four days after culture initiation) are shown in Figure 3A. The significant difference between the seedlings was further confirmed by measurements of the length of coleoptiles, radicles, and lateral seminal roots (Figure 3B). To confirm that observed differences between the transgenic lines were indeed associated with altered JA level, we studied the effect of exogenous JA treatment on the germination of non-transformed Sar-60 seeds. The analysis showed that JA treatment indeed led to delays in seed germination, and the slower growth of coleoptiles and roots (Figure 3C).

The difference in the growth rate between the transgenic plants and the non-transgenic Sar-60 was obvious in soil-grown plants (Figure 4). As seen in Figure 4A,B, the growth of the above-ground parts of Tr-3 and Tr-18 plants was retarded, while Tr-20 plants grew faster. The difference in roots growth was not observed at this growth stage under the given experimental conditions. The difference in plants height was also noticeable during later developmental stages (Figure S2). Further, we found that the flowering time was also affected in the transgenic lines (Figure 4C,D). Anthesis occurred later in Tr-3 plants in comparison to Sar-60, as observed in first, second, and third spikes. As expected, Tr-20 plants flowered earlier.

Leaf senescence symptoms (leaf yellowing) also appear earlier in Tr-20, and later in Tr-3 plants, in comparison to Sar-60. For the quantitative estimation of senescence in the studied plant lines, we have performed the measurements of variable chlorophyll fluorescence parameters, reflecting functional activity of Photosystem II (PS II). The analysis of the functional activity of PS II is a simple and reliable method for the evaluation of leaf senescence when performed with the necessary precautions. It is important to remember that photosynthetic parameters show heterogeneous spatial patterns within the leaf blade, and that individual leaf senescence starts at the tip of the leaf, and then the senescent area enlarges progressively toward the leaf sheath [28]. For the measurements of light-induced chlorophyll fluorescence in leaves of studied plants the area located 10 cm away from the tip of leaf blade was chosen. In preliminary experiments, it has been established that the measured parameter does not differ in young leaves of different genotypes. Results of the analysis confirmed that the senescence time was altered in the studied transgenic lines (Figure 5). Analysis of PS II operating efficiency in the fourth and flag leaves of plants (at maturation stage) in combination with visual assessment (yellowness of leaves) confirmed delayed senescence in Tr-3 and accelerated senescence in Tr-20. Interestingly, detached same-aged leaves of Tr-3 plants also displayed later senescence in comparison to Sar-60 (Figure 5B). Similar trends were observed in independent transgenic lines with altered JA levels: growth and development lines with increased JA levels were slowed down, while in lines with decreased JA level, they were accelerated (Figures S3 and S4). 

### 2.3. *AtOPR3* Overexpression Alters Wheat Tolerance to Freezing Stress

**Figure 6:**
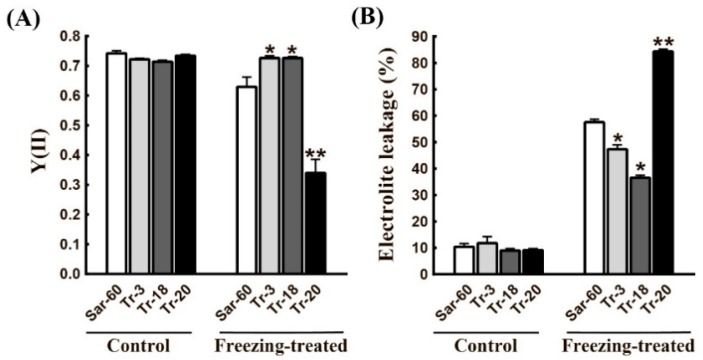
Freezing tolerance test of transgenic wheat plants overexpressing *AtOPR3*. Freezing treatments were performed on homozygous T2 plants, as described in the Materials and Methods section, and then the degree of damage was assessed by measurements of PS II operating efficiency (**A**) and electrolytes leakage (**B**) in control and treated leaves of Sar-60, Tr-3, Tr-18, and Tr-20. Means of 20 measurements ± SE are presented. Stars indicate statistically significant differences from Sar-60 (* *p* ≤ 0.05, ** *p* ≤ 0.001).

To estimate the stress tolerance of transgenic plants with altered JA level, we have performed short-term freezing treatment of plants of different transgenic lines as described in the Materials and Methods section. The freezing-induced damages were estimated by two methods—analysis of PS II activity by measuring light-induced chlorophyll fluorescence parameters and estimation of electrolytes leakage (EL), reflecting the degree of cell membranes damages in leaves. Both analyses showed that stress treatment causes the least amount damage to the leaves of Tr-3 and Tr-18 lines, while leaves of Tr-20 and Tr-15 plants displayed higher sensitivities to given stresses, even in comparison to Sar-60 (Figure 6A,B, and Figure S5). At the same time, no significant differences between the studied lines were observed when plants have been grown under low positive temperature conditions for prolonged periods of time (up to 30 days) (Figure S6).

## 3. Discussion

In this work, we have implemented the transformation of hexaploid bread wheat with the Arabidopsis *OPR3* gene coding for the enzyme catalyzing the reduction of the cyclopentenone ring of 12-OPDA. The expression level of *AtOPR3* varied significantly between different transgenic lines, wherein plants with high *AtOPR3* expression levels have elevated levels of JA. Besides, the expression of *TaAOS*, a jasmonate biosynthesis pathway gene known to be regulated by a positive feedback loop that maintains and boosts JA levels, is also significantly up-regulated in these plants. Increase in *ALLENE OXIDE SYNTHASE* gene expression level in response to the up-regulation of the jasmonate pathway was demonstrated previously [29]. There are three homeologs of *12-OPDA REDUCTASE*, coding for OPRII subgroup proteins, which are potentially involved in the biosynthesis of endogenous jasmonic acid in the wheat genome (one homeolog in each of the A, B, and D subgenomes), TraesCS7A02G412400, TraesCS7B02G311600, and TraesCS7D02G405500. These genes share high similarities at the amino acid level with each other and *AtOPR3* (Figure S7). The expression level of wheat *12-OPDA REDUCTASE* (*TaOPR2*) encoded in the B subgenome, previously shown to be induced by methyl jasmonate [30], was slightly increased in Tr-3, and remained unaltered in other transgenic lines (Figure 2C). We could not detect the expression of *TaOPR2* homeolog genes from the A and D genomes, either because of the low expression level of these genes in leaf tissue, or because of the sequence difference between Saratovskaya-60 and the sequenced wheat varieties. The expression level of the jasmonate receptor COI-1 (*TaCOI-1*) [31] was not altered in transgenic lines (Figure 2C). The gene expression analysis demonstrates that *AtOPR3* gene overexpression selectively affects some genes of the endogenous jasmonate system, while the expression of other genes remains unaltered. The growth and development phenotypes of transgenic wheat plants with high *AtOPR3* expression levels were altered noticeably. These plants are characterized by delayed germination, slower growth, and delayed flowering time and senescence. The obtained results are in accordance with several previously published studies demonstrating the role of jasmonates in the regulation of plant growth and development, including seed germination, root growth, blossoming, fruit ripening, and senescence [13,32,33]. It is known, that JA-mediated suppression of plant growth is implemented through the regulation of cell cycle progression [34]. In Arabidopsis, jasmonate caused a touch-induced rosette diameter reduction and delay, in flowering [35]. The decreased size was also observed in rice plants with increased JA levels caused by the inactivation of the parallel competing hydroperoxide lyase branch of the oxylipin biosynthesis pathway [36]. Transgenic rice plants accumulating high levels of methyl jasmonate (MeJA) as a result of overexpression of the Arabidopsis jasmonic acid carboxyl methyltransferase gene were slightly smaller than the non-transformed controls. Flowering time was not affected in these plants, but grain yield was reduced [37]. Rice mutants *cpm2* (*coleoptile photomorphogenesis2*)/*hebiba* (defective in allene oxide cyclase) and *eg1* (*extra glume1*) (defective in a lipase involved in JA biosynthesis) have longer leaves [38,39]. A rice mutant with low JA levels resulting from a mutation in the *OsAOS1* gene had an accelerated juvenile–adult phase change, and flowered approximately five days earlier than did the wild type [40]. Leaf size was also affected in the rice *aos1* mutant. Increased endogenous JA levels in transgenic soybeans led to alterations in organogenesis: the leaves of the transgenic plants were slightly elongated in length, but they dramatically narrowed in width compared with the non-transformed plants. In addition, the elongation of the primary root was inhibited in the transgenic soybean plantlets, whereas the development of lateral root was stimulated [41]. In *Pharbitis nil*, high concentrations of MeJA inhibited root and shoot growth, while low concentrations of this metabolite, in contrast, enhanced plant growth [42]. MeJA treatment also inhibited growth and flowering in *Chenopodium rubrum* plants [43]. Transgenic potato plants overexpressing the gene coding for jasmonic acid carboxyl methyltransferase, in contrast, showed a significant increase in size and tuber yield [44]. A role for MeJA in the modulation of vernalization and flowering time in einkorn wheat was demonstrated [45]. Bread wheat plants overexpressing the allene oxide cyclase *TaAOC1* developed shorter roots and exhibited enhanced tolerance to salinity [13]. 

We estimated the senescence in growing plants and detached leaves by measuring the activity of PS II. A decline in PS II activity is one of the earliest events during leaf senescence, and visible senescence observed on individual leaves correlates with a decline in PSII activity [46]. Results demonstrate that increased JA levels in wheat plants leads to delay in senescence (brightly manifested in Tr-3), while the decreased level of the hormone leads to earlier senescence (Figure 5). Jasmonate roles in leaf senescence have been studied extensively [47]. Similarly to our observation, JA involvement in the regulation of barley leaf senescence was previously described [48].

As expected, the alteration of JA level also affected wheat tolerance to abiotic stress. It was established in our experiments that Tr-3 and Tr-18 have improved tolerances to short-term freezing treatment (Figure 6), although these plants’ growth at low positive temperatures is not altered (Figure S6). Freezing temperatures are one of the most important abiotic factors controlling the geographical distribution of plants and restricting the use of agricultural lands. Short spring freezing episodes may cause injuries to young wheat plants, affecting the yield of this economically important crop. The role of jasmonates in the regulation of plant responses to abiotic stresses has been established [18,49]. An increase in JA level in response to stress factors was demonstrated for many plant species, including those phylogenetically closely related to wheat barley [50]. Exogenous application of jasmonates also improves plant tolerance to abiotic stresses [51,52]. Numerous studies have shown that jasmonates protect plants from damage caused by low temperatures when it accumulates in tissue or it is applied exogenously [53,54,55,56]. Molecular mechanisms underlying the protective effect of jasmonates have also been characterized. It is known that the transcription pathway *ICE-CBF/DREB1* (Inducer of CBF Expression—C-Repeat Binding Factor/DRE Binding Factor 1), playing a key role in plant adaptation to low temperatures, is regulated by jasmonates [57,58]. 

Interestingly, in two independent transgenic lines, Tr-15 and Tr-20, constitutive expression of *AtOPR3* leads to a decrease in basal JA level in comparison to Sar-60. Gene expression analysis showed that the *AtOPR3* expression level is significantly lower in these plants (about a 40-fold difference between Tr-20 and Tr-3) (Figure 2A). In all assays, these plants displayed phenotypes that were opposite to Tr-3 and Tr-18. Tr-20 showed an earlier germination phenotype, faster growth and development, and higher susceptibility to short-term freezing (Figure 3, Figure 4, Figure 5 and Figure 6). Similar traits, such as faster growth, early senescence, and sensitivity to short-term freezing were also observed in Tr-15 (Figures S3–S5). Currently, we are unable to explain the mechanisms leading to a decrease in JA level upon overexpression of the *AtOPR3* gene, but similar results have been previously obtained in several studies describing transgenic plants overexpressing jasmonates biosynthesis pathway genes [12,59]. Transgenic tobacco plants overexpressing few JA pathway genes from Arabidopsis, including *AtOPR3*, had a decreased level of jasmonate–isoleucine conjugate, the most active metabolite of the pathway [59]. Interestingly, the expression level of jasmonate-regulated genes involved in nicotine biosynthesis was increased in these plants, despite the lack of an increase in the JA–isoleucine conjugate level known to regulate the expression of the mentioned genes. Our previous attempts to manipulate the jasmonate branch via overexpression of *AtAOS* in Arabidopsis led to the silencing of the pathway and the generation of sterile plants [12], a clear physiological response in plants depleted of jasmonates [5,60]. In another research, an attempt to overexpress *AtAOS* in tobacco resulted in the reduction of basal JA level by nearly 10-fold [61]. For transgenic wheat plants overexpressing allene oxide cyclase *TaAOC1*, another gene of the jasmonate biosynthesis pathway, authors reported the increase in wounding-induced levels of JA, while the basal levels of the hormone remained unaltered [13]. The dimerization of OPR proteins leading to the inhibition of enzymatic activity could be another possible explanation for the decrease in JA level upon *AtOPR3* overexpression in wheat, as shown previously for OPR3 from tomato [62]. The jasmonate level in plant tissue is controlled by the intricate regulatory system with positive and negative feedback loops [63,64,65]. Reduction of cyclopentenone catalyzed by AtOPR3 is also a highly regulated process [19]. In wheat, the regulation of the pathway activity may be even more complicated, due to polyploidy [66]. Apparently, jasmonate pathway-related genes are represented by large gene families in hexaploid wheat. According to WheatExp, a homologue-specific database of gene expression profiles [67], there are 12 expressed allene oxide synthase genes; based on the available published data, there are 18 COI genes [68] and 14 JASMONATE-ZIM DOMAIN transcription repressor genes in the wheat genome [69]. Further expression profiling study of the generated transgenic lines may shed light onto the jasmonate-dependent regulation of homolog gene expression in wheat.

Altogether, the results obtained in this study prove that the modulation of jasmonate pathway activity can be used as a tool for the modification of developmental characteristics and tolerance to freezing in wheat. Constitutive *AtOPR3* expression causes a pleiotropic phenotype in transgenic wheat, including the manifestation of beneficial traits and characteristics, which could be disadvantageous to wheat growers. On the one hand, transgenic plants with high *AtOPR3* expression levels are characterized by freezing tolerance and reduced height, which is usually associated with increased resistance against lodging, and enables plants to support high grain yields [70]. On the one hand, these plants display late germination and slower developmental phenotypes, which under field conditions could lead to plant exposure to adverse temperatures and water deficiency during late development [71]. The use of development stage-specific and inducible promoters [72,73] will allow the desired combination of characteristics to be achieved through the fine-tuning of the activity of the jasmonate-based system.

## 4. Materials and Methods 

### 4.1. Plants and Growth Conditions

Non-transgenic and transgenic plants of spring bread wheat ‘Saratovskaya-60’ (Sar-60) (*Triticum aestivum* L. 2*n* = 6*x* = 42, ABD genome) were grown at greenhouse conditions (25 ± 2 °C during the day and 20 ± 2 °C at night). Plants were grown in potted soil under a 16-h photoperiod with additional lighting during the winter period for providing light intensity of up to 150 µmol/m^2^s. For the germination test and segregation analysis, mature seeds or embryos of immature seeds were plated under sterile conditions on Murashige and Skoog medium, or on wet filter paper in a Petri dish. 

### 4.2. Generation of Transgenic Wheat Plants

The full-length coding sequence for the *AtOPR3* gene (AT2G06050, UniProt ID Q9FUP0) (including the sequence for peroxisome targeting signal) was obtained by reverse transcription polymerase chain reaction (RT-PCR) using total RNA isolated from *Arabidopsis thaliana* leaves as a template. *Sma*I and *Sac*I restriction sites were added to gene-specific primer sequences for cloning (Table S2). The *AtOPR3* gene sequence was introduced into pUC19-based vectors between the *Ubi1* promoter from maize and the *Tnos* terminator from *Agrobacterium tumefaciens*. the fragment with the sequence of the target gene with regulatory sequences was then cut out using *Pvu*II, and the PUbi-*AtOPR3*-Tnos fragment was transferred into the *Sma*I site of the psGFP-BAR vector [74], containing two selective marker genes, *GFP* and *BAR*, coding respectively for green fluorescent protein, and phosphinothricin acetyl transferase, conferring resistance to herbicide phosphinotricin. It was established in our previous work that wheat transformation with the psGFP-BAR vector does not result in any phenotypical alterations [27]. For all constructs, sequences were checked after cloning to ensure fidelity and integrity. The generated construct pBAR-GFP.UbiOPR3 was used for biolistic delivery by a particle inflow gun (PIG) into cells of wheat morphogenic callus induced from immature embryos. The vector DNA was bombarded into 377 immature embryo-derived calli of Sar-60 to generate primary transgenic wheat plants (T0). Primary transformants (T0) were selected on medium supplemented with herbicide glufosinate ammonium, and by monitoring the fluorescence of GFP, as described [75]. The herbicide-resistant plantlets with a height of at least 10 cm were transferred to the soil, as described [76], and grown in an environmentally controlled greenhouse, as described in Section 4.1.

Leaf samples collected from plants at heading stage were analyzed for the presence of the insert by PCR. The expression of the transferred genes was confirmed by end-point RT-PCR using RNA isolated from the studied plants as a template. RNA isolation and reverse transcription was carried out as described below (Section 4.4). The primers used for the target sequences amplification are shown in Table S2. 

T1 and T2 progenies of *AtOPR3* overexpressing plants produced by self-pollination of primary T0 transgenic plants were assessed for transgene inheritance. To speed up the segregation analysis and the selection of homozygous transgenic progeny, we analyzed T0→T1→T2 plants, as was described earlier [27,75]. Zygotic T1 embryos were isolated from immature T0 spikes, placed on germination medium for 7–9 days, and then GFP fluorescence was monitored in the seedlings using a ZEISS SteREO Discovery.V12 microscope equipped with a PentaFluar S 120 vertical illuminator. Two commercially available filter sets 38 GFP BP (EX BP 470/40, BS FT 495, EM BP 525/50) and 57 GFP BP (EX BP 470/40, BS FT 495, EM LP 550) (Carl Zeiss MicroImaging GmbH, Jena, Germany) were used to examine GFP expression. Seedlings with a high level of GFP fluorescence were transferred to soil and placed in greenhouse conditions where mature plants were grown. To identify homozygous plants, the pollen from individual mature T1 plants and immature embryos were analyzed for GFP fluorescence (Figure S1A,B), as described previously [27]. Individual homozygous T1 plants were grown in a greenhouse to produce T2 homozygous wheat seeds. T2 homozygous transgenic plants and the T3 progeny obtained from T2 transgenic plants were used for further analysis.

### 4.3. DNA Extraction and PCR Analysis 

Total genomic DNA was extracted from leaf tissues at heading stage, in accordance with the method described [77]. PCR analysis was carried in a thermal cycler, MJ Mini (BIO-RAD, Hercules, CA, USA). Each PCR reaction mixture of 25 µL consisted of 2.5 µL of 10 × buffer for Encyclo DNA polymerase (Evrogen, Moscow, Russia), 0.5 µL of 10 mM dNTPs, 1.25 µL of reverse and forward primers each at 10 µM, 0.5 µL of Encyclo DNA polymerase (Evrogen, Russia), 1 µL DMSO (≥99.5%), 90 ng (1.5 µL) of DNA template, and 16.5 µL of deionized water. Primers are listed in Table S2.

### 4.4. RNA Extraction and Gene Expression Analysis 

Real-time quantitative reverse transcription PCR was used for the analysis of the expression of selected genes: *ALLENE OXIDE SYNTHASE* (*TaAOS*) (GenBank: KJ001800.1), *12-OPDA REDUCTASE 2* (*TaOPR2*) (TraesCS7B02G311600), and *CORONATINE INSENSITIVE 1-LIKE* (*TaCOI*-1) (TraesCS1A02G279100). Gene sequences were obtained from the GenBank and Ensemble Plants databases [78]. Total RNA from the second leaf of T3 plants at the four-leaf stage was isolated by Extract RNA reagent (Evrogen, Moscow, Russia), and further purified using the Clean RNA Standard kit (Evrogen, Moscow, Russia) with on-column DNase I treatment (Thermo Fisher Scientific, Welhem, MA, USA) to eliminate DNA contamination. RNA was reverse-transcribed using the MMLV Reverse Transcriptase kit (Eurogen, Russia). Real-time quantitative PCR was conducted in 20 µL reactions containing complementary DNA (cDNA) synthesized from 10 ng of total RNA, SYBR Green I qPCR Master Mix, (Syntol, Moscow, Russia), and 200 nM for each primer. Amplification was performed using Roche LightCycler® 96 System according to the manufacturer’s instructions. *TaWIN1* and *TaUbi* genes were used as reference genes for the internal controls, as previously described for transcript normalization [26]. Primers are listed in Table S2. 

### 4.5. Jasmonic Acid Extraction and Quantification 

The second leaves of homozygous T3 plants at the four-leaf stage were used for the analysis of JA level. Jasmonic acid extraction was carried out similarly to the procedure previously described [8]. Dihydrojasmonic acid (Merck KGaA, Darmstadt, Germany) was used as an internal standard. The formed methyl ester derivatives of jasmonic acid (JA-ME) and dehydrojasmonic acid were analyzed on a gas chromatograph Bruker 436-GC coupled with a mass spectrometer detector Bruker Scion SQ operating in electron ionization mode. One microliter volume samples were injected by autosampler Bruker CP-8400 in splitless mode at 250 °C, and separated on a column DB-5MS UI (length 30 m, inner diameter 0.25 mm, film thickness 0.25 µm) with helium being used as a carrier gas (constant flow 0.7 mL/min). Oven temperature programming was as follows: hold at 40 °C for 1 min after injection, ramp at 15 °C/min to 150 °C, then increase at 10 °C/min to 250 °C, hold for 10 min. Mass spectral analysis was done in selective ion monitoring mode (SIM). The fragment ions monitored were as follows: JA-ME 224, 151, 83; dihydro-JA-ME 96, 83. The Bruker Workstation 8.2.1. program was used for the data analysis. 

### 4.6. Electrolyte Leakage

The second leaves of homozygous T2 plants at the four-leaf stage were used for the analysis. A total of 2 g of leaves cut into 4 cm pieces were rinsed with deionized water, placed into 50 mL of deionized water, and left on an orbital shaker at room temperature. The conductivity of the solution was measured after 20 h of gentle shaking using the conductivity meter OK-102/1 (Radelkis, Budapest, Hungary). Total conductivity was determined on the same samples after they were autoclaved for 30 min at 121 °C. The samples were cooled down to room temperature prior to measurements. Results were expressed as a percentage of total conductivity.

### 4.7. Chlorophyll Fluorescence Measurements

Light-induced chlorophyll fluorescence measurements were carried out on leaves of growing plants, or on detached leaves, using a FluorPen FP 100 fluorimeter (Photon Systems Instruments, Drasov, Czech Republic). Photosystem II operating efficiency was measured in light-adapted samples according to the FluorPen FP 100 fluorimeter manual, and calculated according to equations: Y(II) = (F’m − Fs)/F’m, where Fs—stationary level and F’m—light-induced maximum level of chlorophyll fluorescence in light-adapted leaves [79]. Taking into account the heterogeneity in photosynthetic parameters within the leaf blade, all measurements were carried out in the area 10 cm away from the tip of the leaf blade. Light-induced chlorophyll fluorescence measurements were performed on the flag and fourth leaves of homozygous T2 plants at the maturation stage to estimate senescence. For the detached leaf senescence analysis, the second leaves of homozygous T2 plants at the four-leaf stage were used.

### 4.8. Exogenous Methyl Jasmonate Treatment

For exogenous methyl jasmonate treatment, 100 µM solution of MeJA was applied to filter paper lining Petri dishes with germinating seeds of Sar-60. 

### 4.9. Freezing-Tolerance Test

Three-week-old homozygous T2 plants at the four-leaf stage growing in pots were placed into a growth chamber at 4 °C for 24 h for cold acclimation. Then, leaves of the low temperature-adapted plants were cut, wrapped into plastic bags, and placed into a chilled vacuum-insulated vessel. Leaves in the vessel were subjected to decreasing temperatures for 30, 60, or 90 min (the temperature was controlled by a wireless thermometer with a remote sensor; the decrease rate was 1 °C/10 min), then leaves in the vacuum-insulated vessel were placed back at 4 °C for another 24 h prior to assessment of leaf damage caused by the freezing treatment by the means of light-induced chlorophyll fluorescence and electrolyte leakage measurements. 

### 4.10. Statistical Analysis

To determine statistically significant differences between genotypes, a one-way analysis of variance (ANOVA) followed by post hoc comparisons by Tukey’s test and the Student’s t-test for independent means were performed.

## Supplementary Materials

Supplementary materials can be found at http://www.mdpi.com/1422-0067/19/12/3989/s1.

## Abbreviations

13-HPOT	13(S)-hydroperoxy-9,11,15-octadecatrienoic acid
12-OPDA	(9S,13S)-12-oxo-phytodienoic acid
JA	Jasmonic acid
MeJA	Methyl jasmonate
