# Supplementary material for: Overexpression of Arabidopsis OPR3 in Hexaploid Wheat (Triticum aestivum L.) Alters Plant Development and Freezing Tolerance
Source: Int J Mol Sci. 2018 Dec 11;19(12):3989. doi: 10.3390/ijms19123989 (PMC6320827; doi:10.3390/ijms19123989)
Supplement: Supplementary file 1 [file ijms-19-03989-s001.zip › Supplemental Table 1.docx]

Supplemental Table 1. Endogenous level of JA in leaves of studied transgenic lines and Sar-60.

| **Line** | **Jasmonic acid,** (ng/g f.w.) | **Phenotype** |
| --- | --- | --- |
| **Sar-60** | 3.04 ± 0.13 |  |
| **Tr-3** | 5.17 ± 0.46* | Slow growing |
| **Tr-18** | 3.68 ± 0.17 | Slow growing |
| **Tr-15** | 2.44 ± 0.11* | Fast growing |
| **Tr-20** | 2.33 ± 0.08* | Fast growing |

* Statistically significant difference in comparison to Sar-60, p ≤ 0.05
