# Supplementary material for: Overexpression of Arabidopsis OPR3 in Hexaploid Wheat (Triticum aestivum L.) Alters Plant Development and Freezing Tolerance
Source: Int J Mol Sci. 2018 Dec 11;19(12):3989. doi: 10.3390/ijms19123989 (PMC6320827; doi:10.3390/ijms19123989)
Supplement: Supplementary file 1 [file ijms-19-03989-s001.zip › Supplemental Table 2.docx]

Supplemental Table 2. List of primers.

| **Analysis** | **Gene** | | **Primers** | **Product size, bp** | |
| --- | --- | --- | --- | --- | --- |
| Cloning | *AtOPR3^*^* | 5'-AATT**CCCGGG**GACATGACGGCGGCACAAGG-3'  5'-ATCG**GAGCTC**ACTCAGAGGCGGGAAAAAGGAGC-3' | | 1201 |  |
| Genotyping | *AtOPR3* | 5'-CCGAAGGCACCATGGTCTCTCCC-3'  5'-TCGGAAGCTTCTAAAGCCCGAGG-3' | | 295 |  |
|  | *GFP* | 5'-GCGACGTAAACGGCCACAAG-3'  5'-CCAGCA GGACCATCTGTGATCG-3' | | 600 |  |
|  | *TaWIN1* | 5'-TTTTCTGTGTTCTACTATGAGATCTTGAA-3'  5'-AAGTGCATAATTAAACAGAGGTAGTGATG-3' | | 348 |  |
| qPCR | *AtOPR3* | 5'-CCGAAGGCACCATGGTCTCTCCC-3'  5'-TCGGAAGCTTCTAAAGCCCGAGG-3' | | 295 |  |
|  | *TaAOS* | 5'-CAAGGCCGACATGAACATCGAGA-3'  5'-GACGCCGGTGAATTCAACCTTG-3' | | 355 |  |
|  | *TaOPR2* | 5'-AGCAGGCTTCGATGGCATTGAGAT-3'  5'-GCGATTTAGGCTTGCCGAGGAAC-3' | | 683 |  |
|  | *TaCOI1* | 5'-GCTTTGCTCCTTCGGACTTAC-3'  5'-CGACAACCCCCAATCCTCTA-3' | | 199 | |
|  | *TaWIN1* | 5'-TTTTCTGTGTTCTACTATGAGATCTTGAA-3'  5'-AAGTGCATAATTAAACAGAGGTAGTGATG-3' | | 348 | |
|  | *TaUbi* | 5'-AAACCCTCACTGGCAAGACC-3'  5'-GCACCAAACCACAGGACTCG-3' | | 349 | |

* Underlined nucleotides in primers’ sequences are SmaI and SacI restriction sites.
