# Supplementary material for: Overexpression of Arabidopsis OPR3 in Hexaploid Wheat (Triticum aestivum L.) Alters Plant Development and Freezing Tolerance
Source: Int J Mol Sci. 2018 Dec 11;19(12):3989. doi: 10.3390/ijms19123989 (PMC6320827; doi:10.3390/ijms19123989)
Supplement: Supplementary file 1 [file ijms-19-03989-s001.zip › Supplementary figures 2018 12 06.docx]

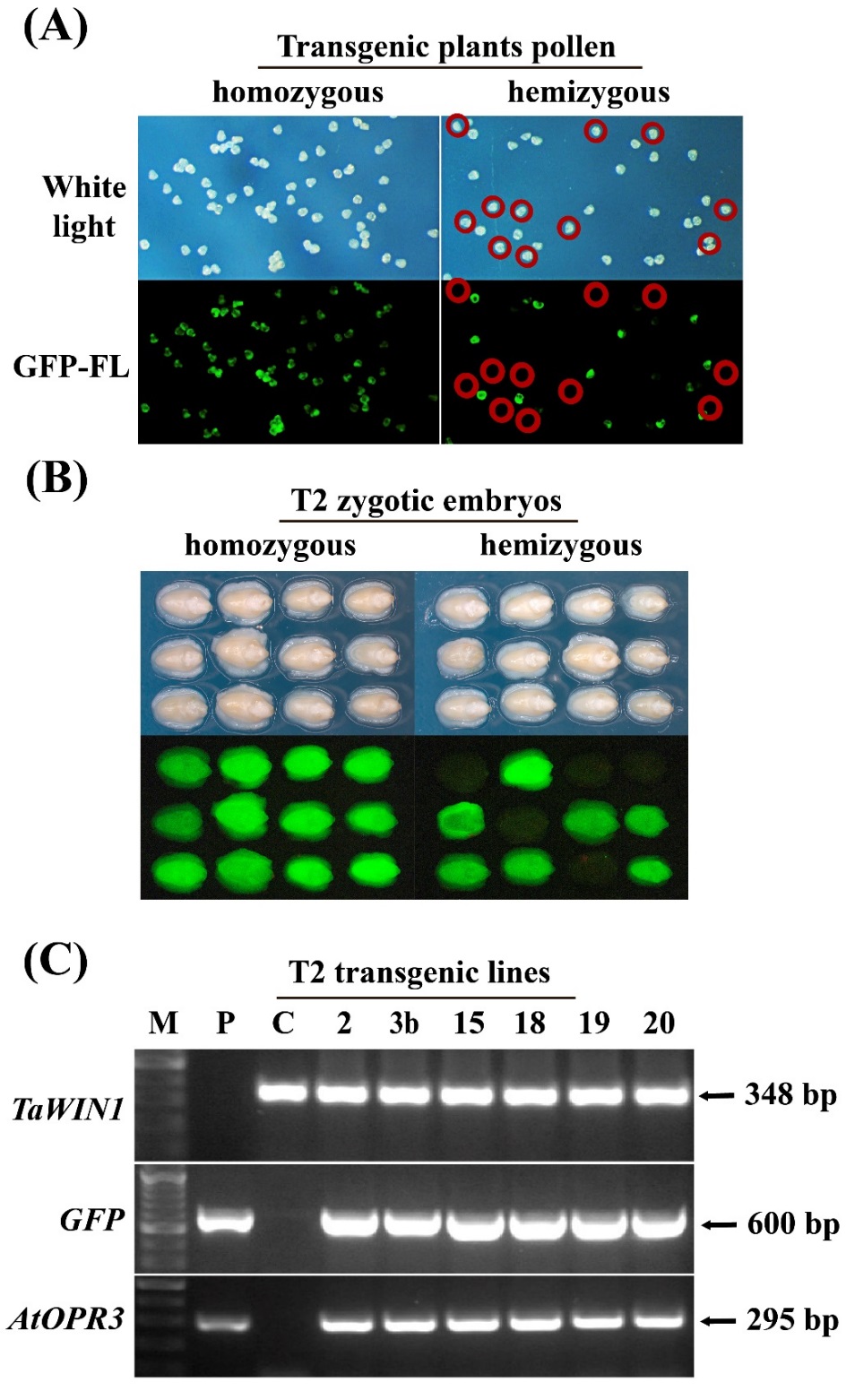


**Supplemental Figure 1.** Analyses of homozygous transgenic wheat plants (T2) selected after self-pollination of T0→T1 transgenic plants transformed with pBAR-GFP.UbiOPR3.

(**A**) Analysis of GFP fluorescence in the pollen of transgenic T2 plants, homozygous (left panels) and hemizygous (right panels). (**B**) Analysis of GFP fluorescence in the T2 zygotic embryos. (**C**) End-point RT-PCR analysis on total RNA of selected homozygous transgenic T2 populations for the expression of reference gene *TaWIN1* (upper panel), *GFP* gene (middle panel) and *AtOPR3* (bottom panel). Lane M, DNA ladder as molecular weight marker; Lane P, plasmid DNA pBAR-GFP.UbiOPR3; Lane C, non-transgenic wheat plant ‘Saratovskaya-60’; Lanes represents homozygous progenies for the primary transgenic wheat plants Tr-2, Tr-3b, Tr-15, Tr-18, Tr-19 and Tr-20.


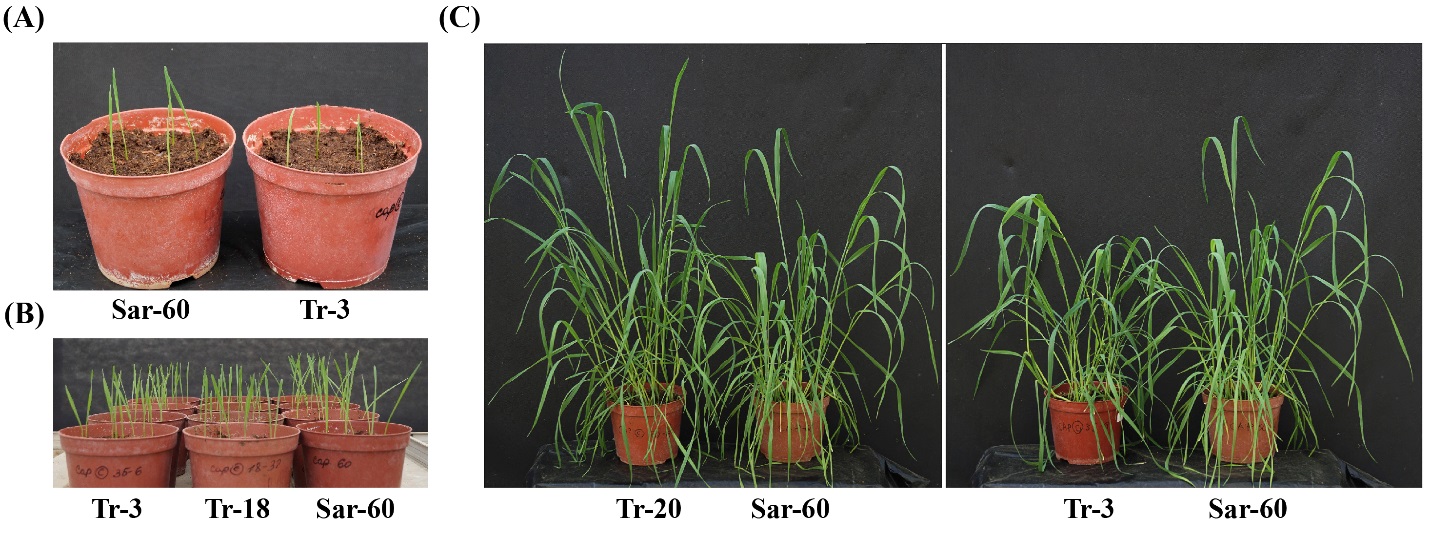


**Supplemental Figure 2.** Transgenic T2 wheat plants overexpressing *AtOPR3* gene have altered growth phenotype. The pictures of plants growing in pots for four (**A**) and seven days (**B**) after the sowing. (C) The pictures of soil-grown tree-week-old plants: Tr-20 in comparison to Sar-60 (left side), and Tr-3 in comparison to Sar-60 (right side).


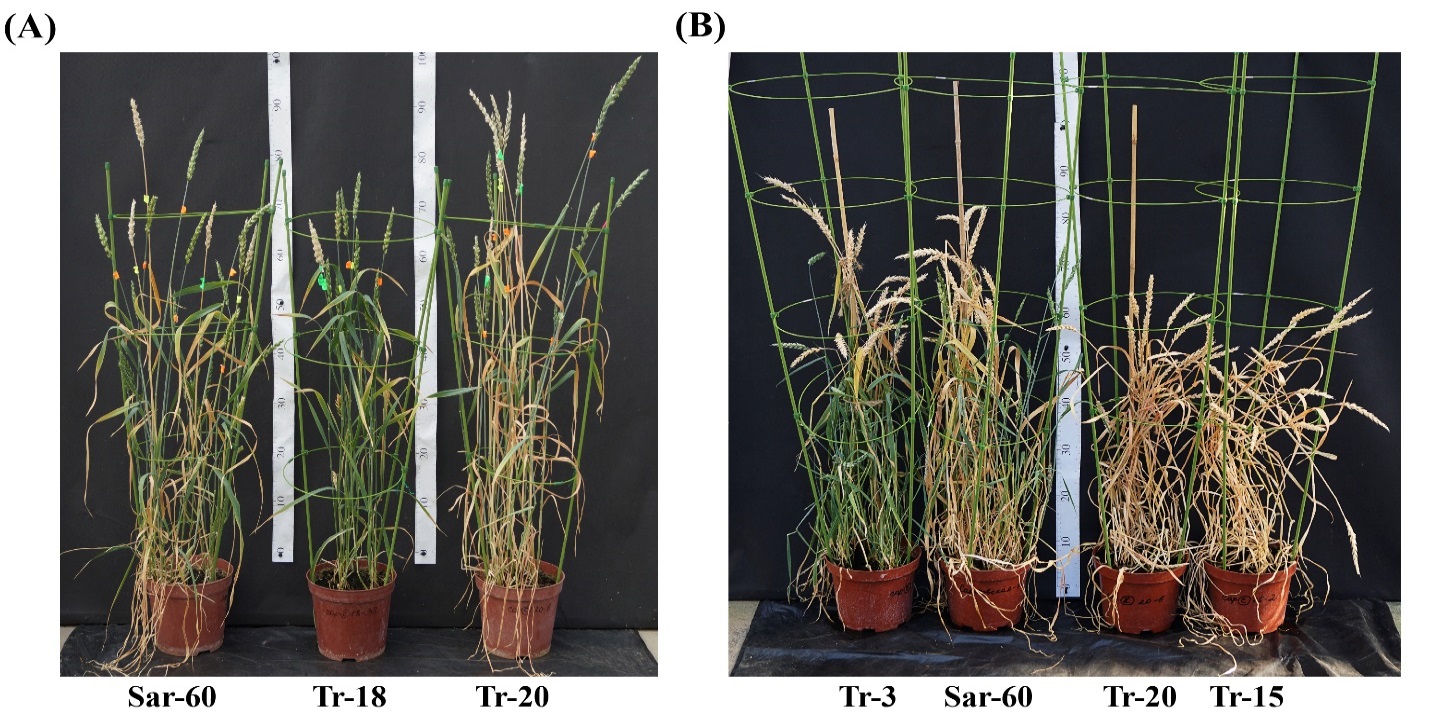


**Supplemental Figure 3.** Transgenic wheat plants overexpressing *AtOPR3* gene have altered growth and development phenotype. The pictures of 70-day-old (A) and 90-day-old (B) plants, Sar-60, Tr-3, Tr-15, Tr-18 and Tr-20 growing in pots.


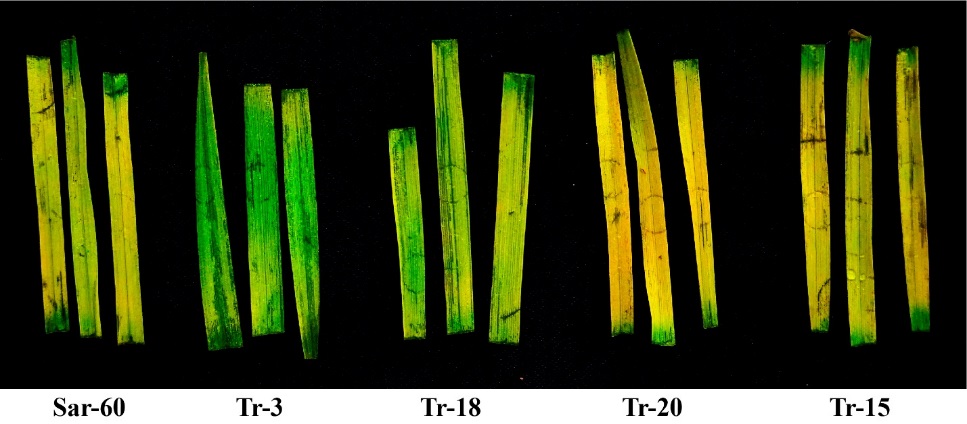


**Supplemental Figure 4.** The senescence of detached leaves is altered in wheat plants overexpressing *AtOPR3.* Same-aged detached leaves of Sar-60, Tr-3, Tr-15, Tr-18, and Tr-20 were kept on water in the dark. The picture was taken on the fifth day of the experiment.


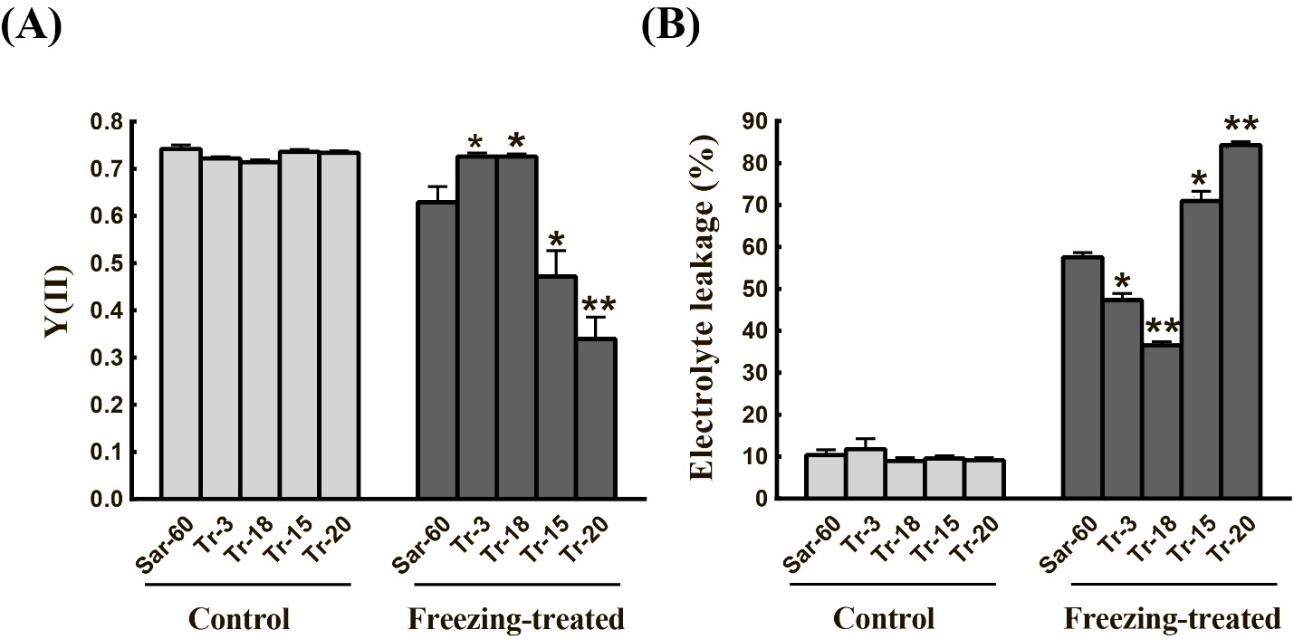


**Supplemental Figure 5.** Freezing tolerance test of transgenic wheat plants overexpressing *AtOPR3*.

Freezing treatment was performed as described in Materials and Methods section, and then the degree of damages was assessed by measurements of PS II operating efficiency (A) and electrolytes leakage (B) in control and treated leaves of Sar-60, Tr-3, Tr-18, Tr-15 and Tr-20. Means of 20 measurements ±se are presented. Stars indicate statistically significant difference from Sar-60 (* p ≤ 0.05, ** p ≤ 0.001).


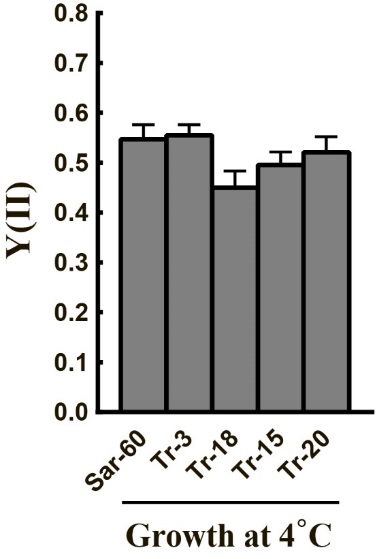


**Supplemental Figure 6.** Sar-60 and transgenic wheat plants overexpressing *AtOPR3* were germinated under greenhouse conditions and placed for 1 month to growth chamber where temperature 4-6 ºC was maintained. The operating efficiency of PS II was measured in 10 plants of each genotype as it is described in Materials and Methods section.


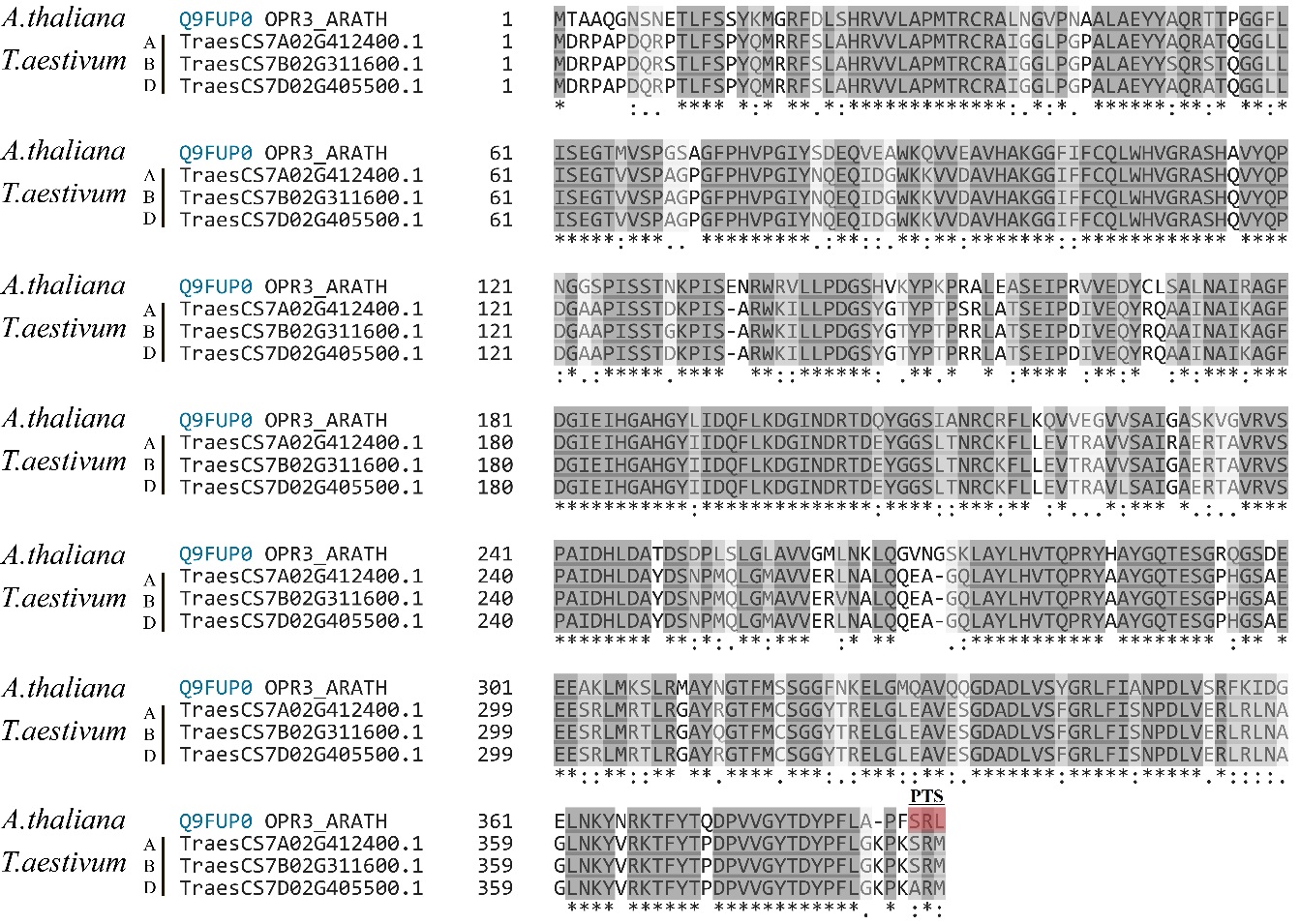


**Supplemental Figure 7.** Alignment of amino acid sequences of Arabidopsis OPR3 and putative OPRII enzymes from *T. aestivum*. Amino acid sequence alignment was generated with Clustal Omega. The symbols below the alignment indicate complete conservation (*), sites with strongly conserved properties (:), and sites with weakly conserved properties (.). The sequences are numbered from the N-terminus of the mature proteins. “PTS” above the alignment indicates peroxisomal targeting signal.

Supplemental Table 1. Endogenous level of JA in leaves of studied transgenic lines and Sar-60.

| **Line** | **Jasmonic acid,** (ng/g f.w.) | **Phenotype** |
| --- | --- | --- |
| **Sar-60** | 3.04 ± 0.13 |  |
| **Tr-3** | 5.17 ± 0.46* | Slow growing |
| **Tr-18** | 3.68 ± 0.17 | Slow growing |
| **Tr-15** | 2.44 ± 0.11* | Fast growing |
| **Tr-20** | 2.33 ± 0.08* | Fast growing |

* Statistically significant difference in comparison to Sar-60, p ≤ 0.05

Supplemental Table 2. List of primers.

| **Analysis** | **Gene** | | **Primers** | **Product size, bp** | |
| --- | --- | --- | --- | --- | --- |
| Cloning | *AtOPR3^*^* | 5'-AATT**CCCGGG**GACATGACGGCGGCACAAGG-3'  5'-ATCG**GAGCTC**ACTCAGAGGCGGGAAAAAGGAGC-3' | | 1201 |  |
| Genotyping | *AtOPR3* | 5'-CCGAAGGCACCATGGTCTCTCCC-3'  5'-TCGGAAGCTTCTAAAGCCCGAGG-3' | | 295 |  |
|  | *GFP* | 5'-GCGACGTAAACGGCCACAAG-3'  5'-CCAGCA GGACCATCTGTGATCG-3' | | 600 |  |
|  | *TaWIN1* | 5'-TTTTCTGTGTTCTACTATGAGATCTTGAA-3'  5'-AAGTGCATAATTAAACAGAGGTAGTGATG-3' | | 348 |  |
| qPCR | *AtOPR3* | 5'-CCGAAGGCACCATGGTCTCTCCC-3'  5'-TCGGAAGCTTCTAAAGCCCGAGG-3' | | 295 |  |
|  | *TaAOS* | 5'-CAAGGCCGACATGAACATCGAGA-3'  5'-GACGCCGGTGAATTCAACCTTG-3' | | 355 |  |
|  | *TaOPR2* | 5'-AGCAGGCTTCGATGGCATTGAGAT-3'  5'-GCGATTTAGGCTTGCCGAGGAAC-3' | | 683 |  |
|  | *TaCOI1* | 5'-GCTTTGCTCCTTCGGACTTAC-3'  5'-CGACAACCCCCAATCCTCTA-3' | | 199 | |
|  | *TaWIN1* | 5'-TTTTCTGTGTTCTACTATGAGATCTTGAA-3'  5'-AAGTGCATAATTAAACAGAGGTAGTGATG-3' | | 348 | |
|  | *TaUbi* | 5'-AAACCCTCACTGGCAAGACC-3'  5'-GCACCAAACCACAGGACTCG-3' | | 349 | |

* Underlined nucleotides in primers’ sequences are SmaI and SacI restriction sites.
