# Supplementary figures and images for: Overexpression of Arabidopsis OPR3 in Hexaploid Wheat (Triticum aestivum L.) Alters Plant Development and Freezing Tolerance
Source: Int J Mol Sci. 2018 Dec 11;19(12):3989. doi: 10.3390/ijms19123989 (PMC6320827; doi:10.3390/ijms19123989)

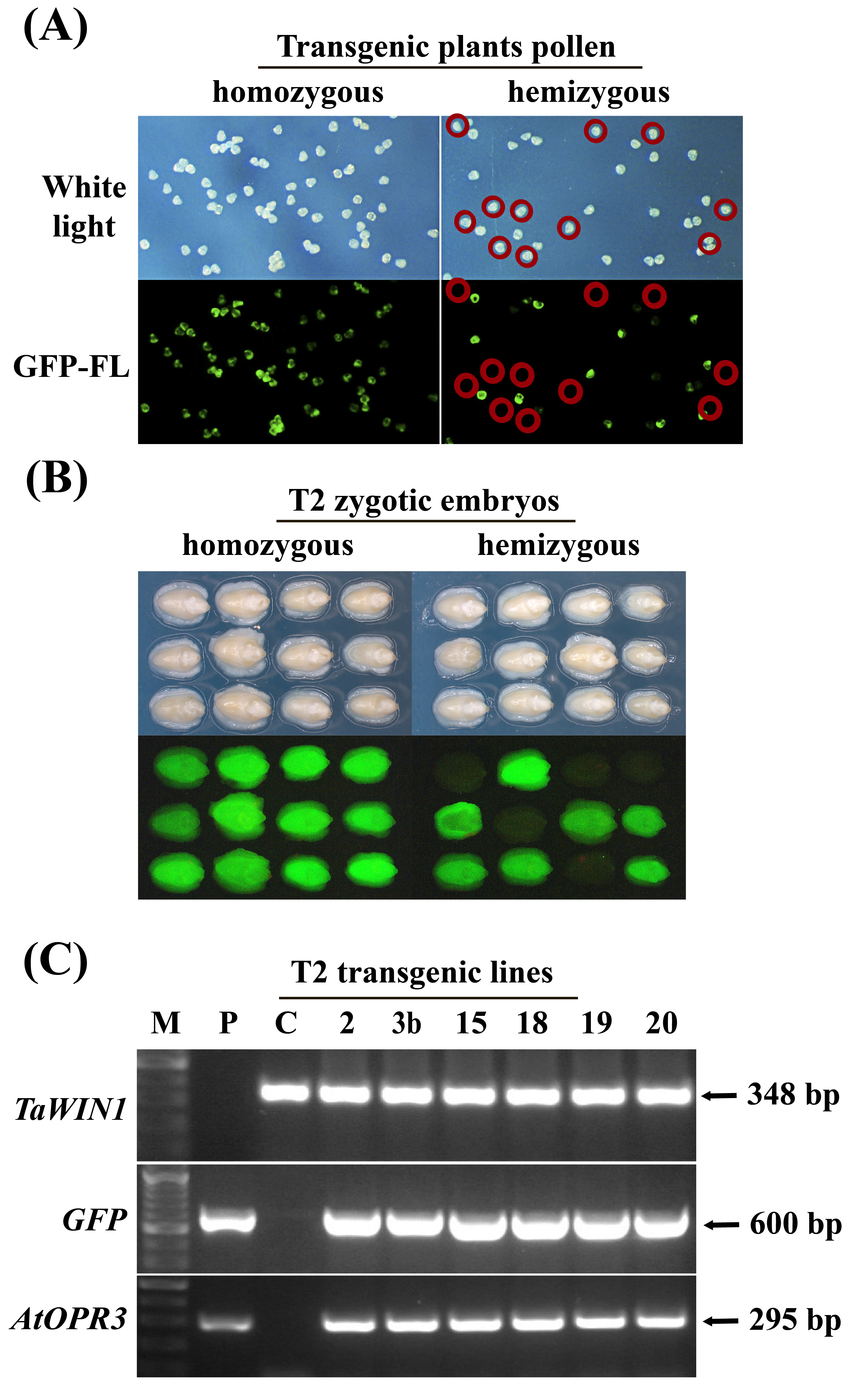

Supplement: Supplementary file 1 [file ijms-19-03989-s001.zip › Suppl. 1.jpg]

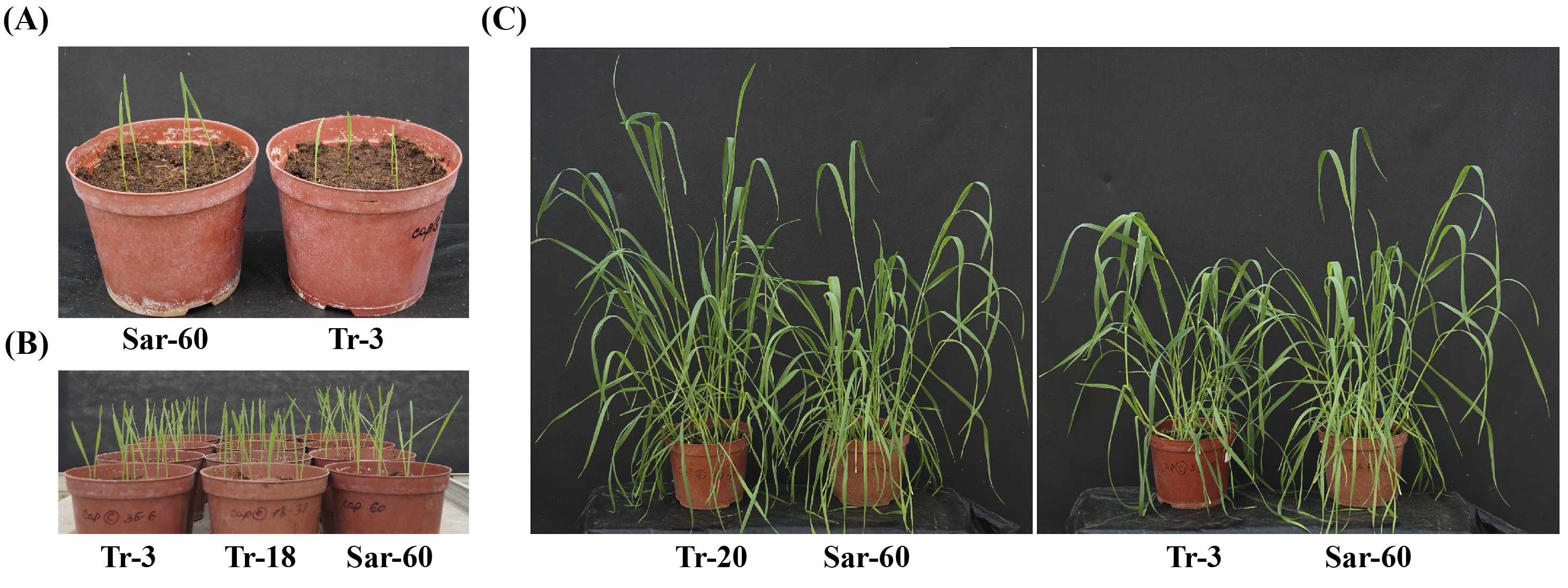

Supplement: Supplementary file 1 [file ijms-19-03989-s001.zip › Suppl. 2.jpg]

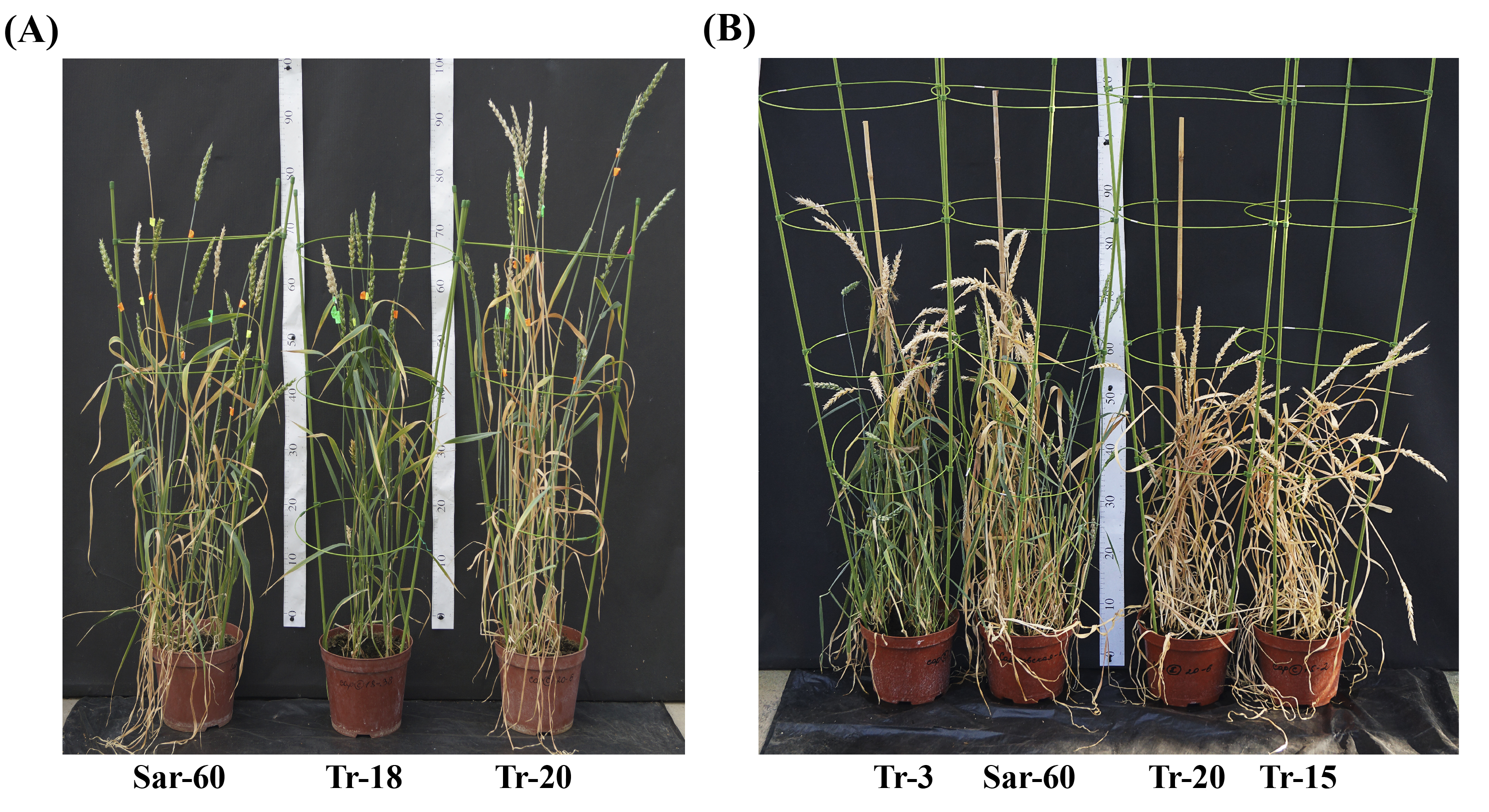

Supplement: Supplementary file 1 [file ijms-19-03989-s001.zip › Suppl. 3.jpg]

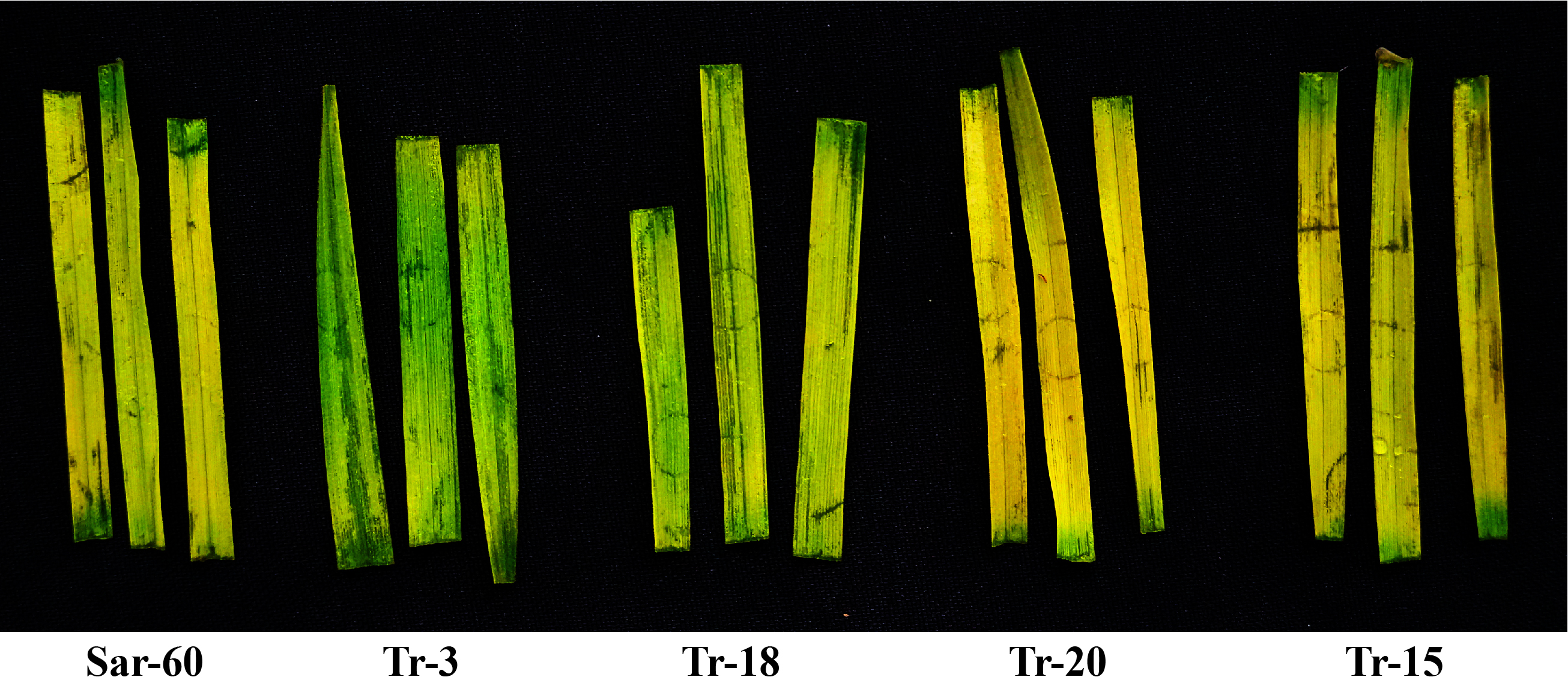

Supplement: Supplementary file 1 [file ijms-19-03989-s001.zip › Suppl. 4.jpg]

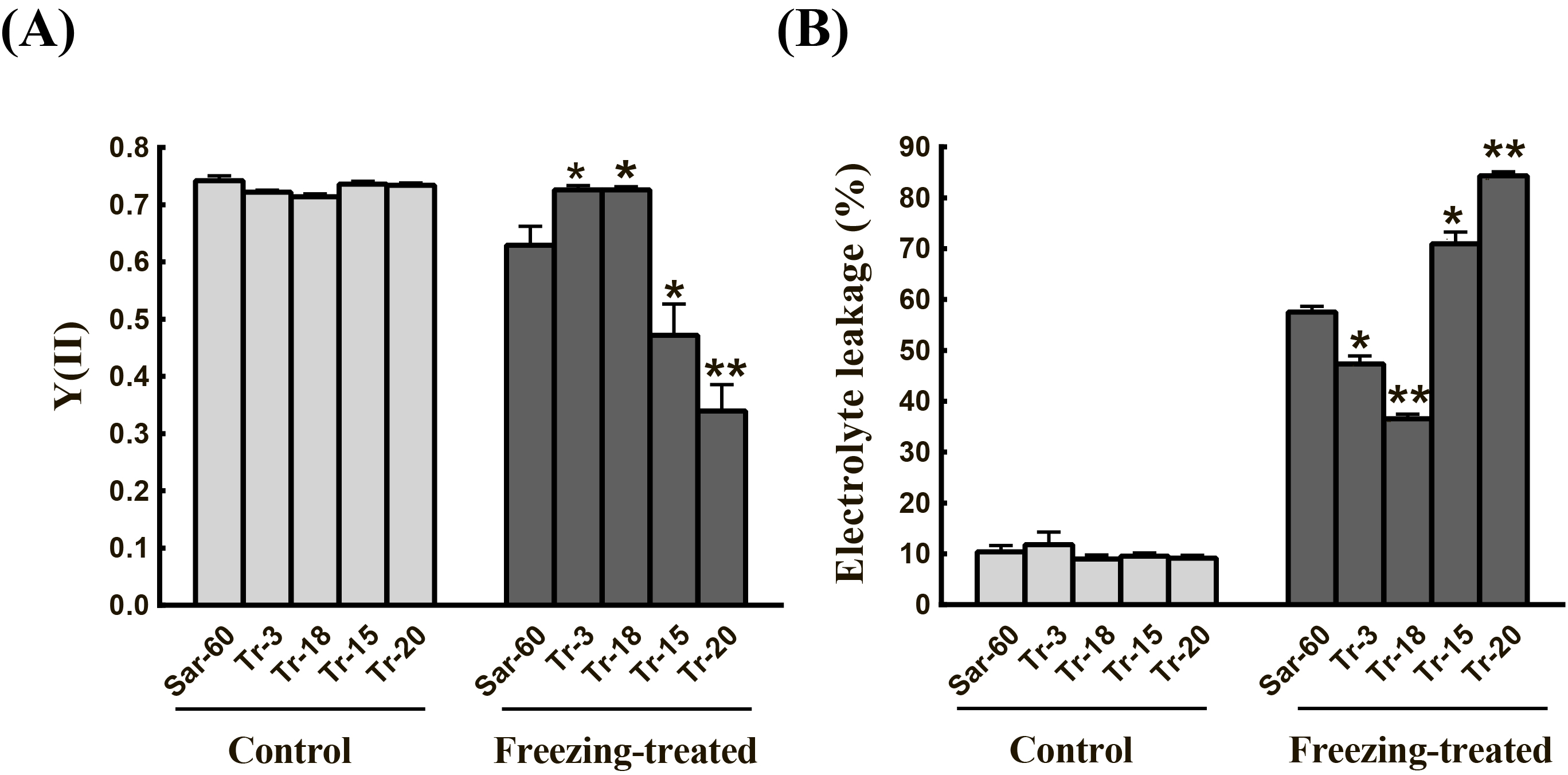

Supplement: Supplementary file 1 [file ijms-19-03989-s001.zip › Suppl. 5.jpg]

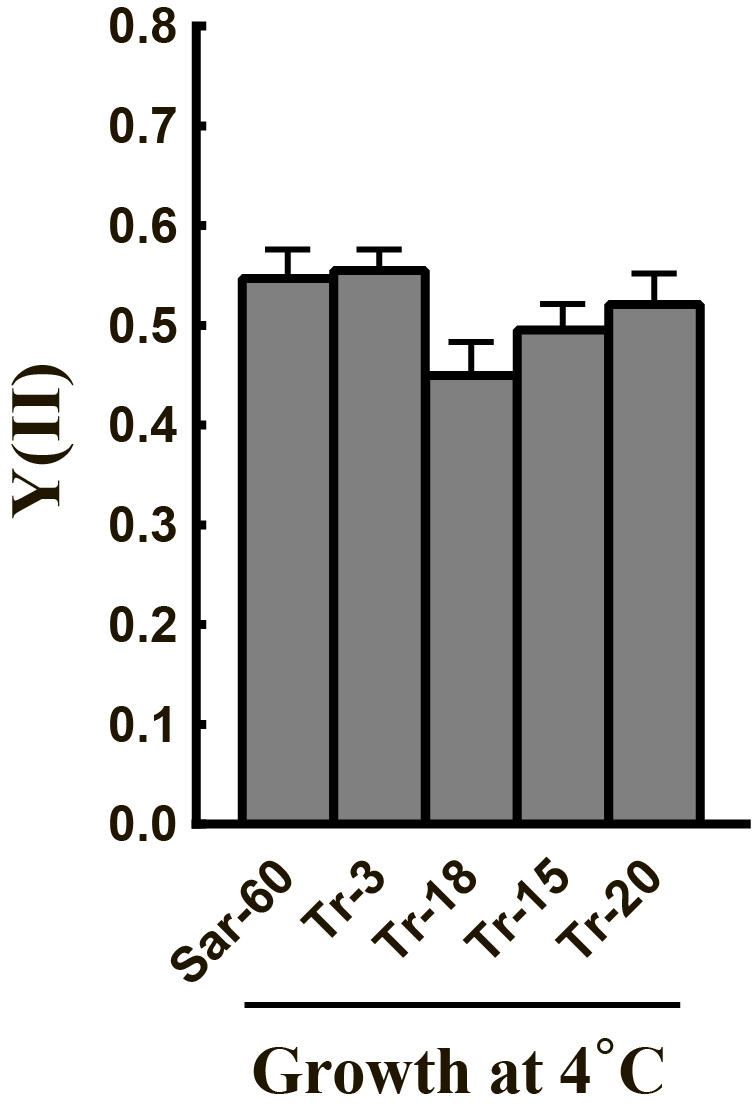

Supplement: Supplementary file 1 [file ijms-19-03989-s001.zip › Suppl. 6.jpg]

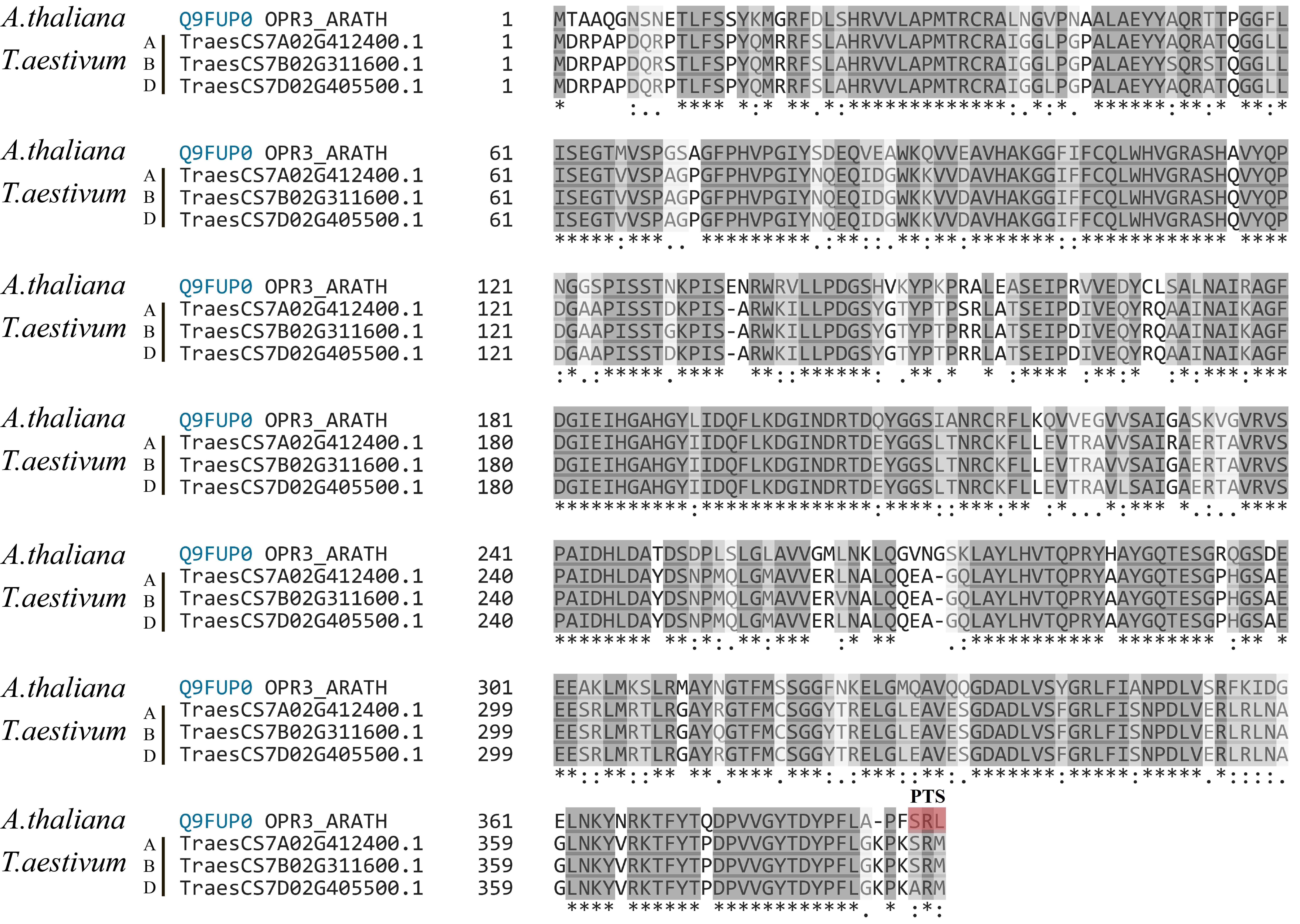

Supplement: Supplementary file 1 [file ijms-19-03989-s001.zip › Suppl. 7.jpg]
